# Modeling Zero‐Gap Saltwater Electrolysis With Advective Flow Through a Thin‐Film Composite Membrane

**DOI:** 10.1002/cssc.202501310

**Published:** 2026-02-08

**Authors:** Rachel F. Taylor, Chenghan Xie, Bin Bian, Amir Akbari, Bruce E. Logan

**Affiliations:** ^1^ Department of Chemical Engineering Pennsylvania State University University Park Pennsylvania USA; ^2^ Department of Civil and Environmental Engineering Pennsylvania State University University Park Pennsylvania USA

**Keywords:** convective flow, green hydrogen production, saltwater electrolysis, thin‐film composite membranes, zero‐gap flow cell

## Abstract

In zero‐gap saltwater electrolysis, ion transport is influenced by convective forces, but their effects have not been examined when using thin‐film composite (TFC) membranes with advective flow through the membrane. In this study, we adapted a one‐dimensional solution‐friction transport model for a zero‐gap electrolyzer to incorporate measured water flux across a TFC membrane. Open‐circuit or electrolysis (20 mA cm^–2^) experiments quantified ion transport with and without electrochemical reactions. Water velocity, estimated from volume changes in the anolyte and the catholyte, was used to infer convective contributions to ion transport. Ion‐specific friction coefficients were determined using open‐circuit data. Using the fitted friction factors and incorporating water flux, the modeled ion crossover concentration showed good agreement with electrolysis data, including changes caused by reversing the membrane orientation. Removing the convective flux from the model showed up to a 740% change in predicted ion crossover and worsened agreement with experimental data. The strong correlation between the fraction of charge carried by major salt ions and the measured water flux suggests that electroosmotic drag could be one of the main mechanisms responsible for the observed water flux. These results highlight the importance of incorporating solution convection when modeling ion behavior in zero‐gap systems using TFC membranes.

## Graphical abstract

During saltwater electrolysis with a thin‐film composite membrane, ion and water transport are coupled. The direction of water flux follows that of the dominant salt ion carrying charge. When Na^+^ transport from the anode to the cathode is dominant, fluid flow occurs in the same direction. Reversing the membrane orientation shifts NO_3_– to be the major salt charge carrier from the cathode to the anode, with fluid flow in the same direction.

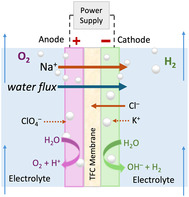

## 1 Introduction

Hydrogen production predominantly relies on fossil fuels, and in 2023 alone, it resulted in the release of 920 million tons of carbon dioxide into the atmosphere, emphasizing the urgent need for efficient, cost‐effective, and carbon‐neutral electrolysis technologies that enable green hydrogen production [1]. Conventional water electrolysis methods, including polymer exchange membrane (PEM) electrolysis and alkaline water electrolysis (AWE), suffer from high capital costs or low efficiencies, an issue highlighted across multiple studies [2, 3, 4]. In addition, their reliance on ultrapure water, which can add complexity to water electrolyzer process trains and be costly to produce, is another challenge for widespread use of these technologies [5]. To minimize the capital and operational costs associated with water purification, direct electrolysis of saltwater is being researched as an alternative to ultrapure water [6, 7].

Directly electrolyzing saltwater faces several challenges. In unbuffered saltwater (pH ∼ 8), salt ions compete with water ions to carry charge, causing large pH gradients across the separator or membrane [6, 8]. Additionally, chloride ions (Cl^–^) in saltwater can oxidize at the anode to form chlorine gas and other oxidized chlorine species (e.g., HClO^–^), which can damage electrolyzer components and shorten the system lifespan [9, 10]. To mitigate Cl^–^ oxidation at the anode, thin‐film composite (TFC) membranes have been proposed to separate a saltwater catholyte from an anolyte composed of a fully oxidized salt solution (e.g., NaClO_4_), which prevents Cl^–^ oxidation while maintaining a high electrolyte conductivity [8, 11].

TFC membranes that were primarily developed for reverse osmosis (RO) desalination of salty water [12, 13] have only recently been studied for electrochemical applications [8, 14]. Compared to conventional PEMs, TFC membranes are an affordable (∼$10/m^2^) and relatively sustainable alternative to membranes using fluorinated compounds [8, 15]. TFC membranes can have comparable resistances to PEMs (6.1 ± 0.1 Ω cm^2^ for a brackish water RO membrane vs. 7.2 ± 0.8 Ω cm^2^ for Nafion 117) and similar required electrolysis potentials in saltwater systems (3.5 V for a TFC membrane and Nafion during electrolysis at 40 mA cm^–2^) [8, 11]. Removing the polyester web support layer on typical TFC membranes helps to reduce resistance, and selective coatings can even further reduce chloride ion transport through the membrane to the anode [16, 17].

To better understand ion transport in different systems using TFC membranes, researchers have used solution‐friction transport modeling to predict salt and water ion transport across these membranes [18, 19]. Modeling enables quantification of the location of the water association reaction within the TFC membrane and provides insights into factors controlling competitive ion transport [18, 19]. Initial modeling efforts focused on bench‐scale electrolysis cells with large electrolyte volumes separating the electrodes from the membrane, and did not incorporate convective flow effects [18, 19]. However, in zero‐gap electrolysis systems used industrially, additional transport phenomena—such as electroosmotic flow—can drive water movement between electrolyte compartments [20, 21]. In a recent study, significant water transport was observed from the anolyte to the catholyte when using TFC membranes with an alkaline anolyte and saltwater catholyte in a zero‐gap configuration [14]. The impact of convective electrolyte flow on ion transport or system performance in that study was not further examined.

In this study, we used solution‐friction modeling for the zero‐gap flow cell geometry rather than widely spaced electrodes and modified it to investigate how convective flow through the membrane impacted salt and water ion transport. To examine the utility of the model for electrolyzers, we measured ion crossover concentrations over 6 h under both open‐circuit and electrolysis (20 mA cm^–2^) conditions. Open‐circuit data were used to fit ion‐specific friction factors in the solution‐friction model, while average water velocity across the membrane was estimated from measured volume changes in each compartment and incorporated into the model. We then examined how membrane orientation, with the active layer of the TFC membrane facing the anode or cathode, influenced water transport. With this model, we calculated the fraction of ion transport attributable to convection. Both nitrate (NO_3_^–^) and chloride (Cl^–^) were studied in 0.6 M KNO_3_ and 0.6 M KCl electrolytes to compare their transport behavior and infer the extent of chloride oxidation at the anode under the different experimental conditions.

## 2 Experimental Materials and Methods

### 2.1 Materials

ACS‐grade sodium perchlorate (NaClO_4_) (anhydrous, 98%−102%, Thermo Scientific), sodium nitrate (NaNO_3_) (≥99.0%, Sigma Aldrich), sodium chloride (NaCl) (≥99.0%, Sigma Aldrich), potassium nitrate (KNO_3_) (≥99.0%, Sigma Aldrich), and potassium chloride (KCl) (99.0%−100.5%, VWR Chemicals) were used to prepare the electrolytes for the flow cell experiments. All solutions were made using deionized (DI) water (>18.2 mΩ cm) made with a Milli‐Q ultrapure water purification system (Synergy).

Flat‐sheet SW30XLE TFC membranes (DOW chemical) were used in all experiments. Prior to use, the membranes were cut into 3 cm × 3 cm pieces and hydrated in a 25% (v/v) isopropanol solution for 30 min to remove any trapped air bubbles. They were then rinsed with DI water thoroughly and stored in DI water at 4°C.

Carbon cloth (2 cm × 2 cm) served as the electrode backbone. To enhance hydrophilicity, the cloth was heated at 450°C for 24 h before being coated with the catalyst. The resulting carbon cloth had increased wettability without any mechanical degradation. A slurry of carbon‐supported platinum (Pt) catalyst was prepared and painted onto the carbon cloth with a brush. Each electrode slurry contained 33 µL DI water, 133 µL isopropanol, 267 µL 5% Nafion solution (Ion Power, Pennsylvania), and 40 mg of 10% Pt/C powder, resulting in 1 mg/cm^2^ of Pt on each electrode. The slurry was stirred for 1 h before being applied to the carbon cloth. The coated electrodes were air‐dried overnight before use in the flow experiments.

### 2.2 Zero‐Gap Flow Cell Experiments

**FIGURE 1:**
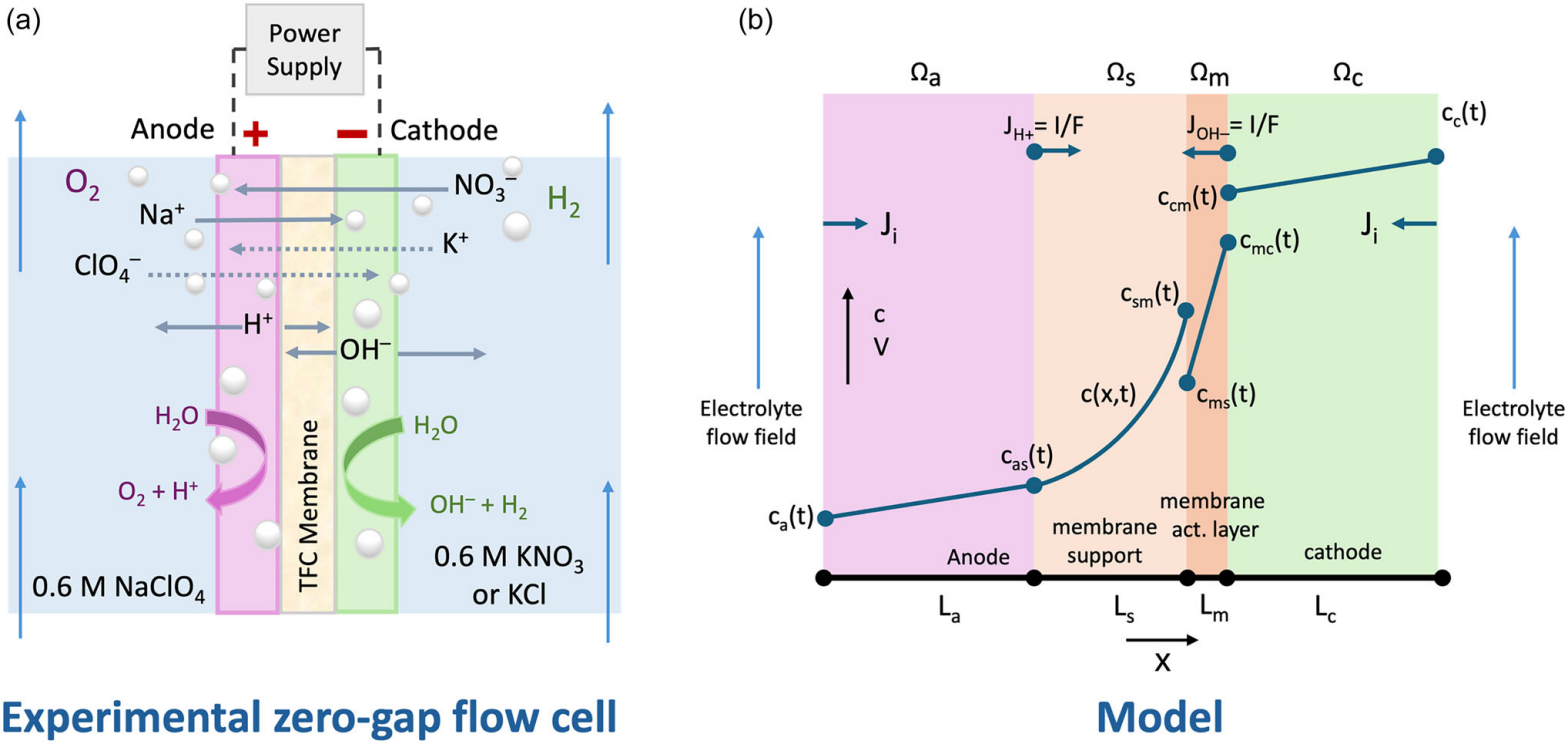
(a) Depiction of a small cross‐section of zero‐gap electrolyzer stack used for saltwater electrolysis with a thin‐film composite membrane. (b) Schematic of the 1‐D model geometry used to simulate the zero‐gap saltwater electrolysis.

Ion transport across the TFC membrane was studied in a zero‐gap flow cell (Scribner, North Carolina). The electrolyzer was assembled with a graphite cathodic current collector and a titanium anodic current collector with serpentine flow fields (Figure S1). The anode and cathode were placed adjacent to the flow field, with the catalyst facing the membrane, secured in place by plastic spacers (237 µm). A hydrated TFC membrane was sandwiched between the electrodes, with the active‐layer orientation (facing the anode or cathode) varied depending on the experiment (Figure 1a). The stack was secured with bolts that were tightened to 9 N m using a torque wrench. Electrolytes (100 mL) were recirculated at 25 mL min^–1^ using a peristaltic pump for 6 h. Samples (1 mL) were collected from the anolyte and catholyte at the start and every hour after. At the end of all experiments, the volumes in the anolyte and catholyte were measured.

In saltwater, Cl^–^ is the dominant anion, but modeling its transport during electrolysis is challenging due to potential Cl^–^ oxidation at the anode in the experiments and the resulting mass balance discrepancies. Nitrate (NO_3_^–^) has previously been used as a nonreactive surrogate for Cl^–^ in modeling studies to avoid these issues [18]. However, NO_3_^–^ has been shown to permeate TFC membranes more readily than Cl^–^ under desalination conditions, due to hydration energy, diffusivity, and partitioning effects [22, 23]. Therefore, transport comparisons between NO_3_^–^ and Cl^–^ are qualitative. In this study, both anions are used: NO_3_^–^ enables reliable model validation and mass balance closure, while Cl^–^ provides a more realistic representation of anion transport during saltwater electrolysis.

Ion diffusion across the membrane and electrodes was first studied without applied current (open‐circuit mode). To isolate the transport behavior of individual ions, either the cation or anion was held constant while the counter‐ion was varied between the anolyte and catholyte. For anion transport experiments, Na^+^ was used as the common cation in both compartments. Using NaNO_3_ in the catholyte and NaClO_4_ in the anolyte enabled comparison of NO_3_^–^ and ClO_4_^–^ transport. Using NaCl in the catholyte and NaClO_4_ in the anolyte enabled comparison of Cl^–^ and ClO_4_^–^ transport. To compare cation transport, NO_3_^–^ was used as the common anion, with Na^+^ in the anolyte and K^+^ in the catholyte. The concentration of all solutions was set to 0.6 M to mimic the concentration of seawater. The active layer orientation was set to face the catholyte in all open‐circuit experiments, consistent with the orientation used in previous saltwater electrolysis experimentation and modeling [16, 18].

To examine ion transport during electrolysis, a current density of 20 mA cm^–2^ was applied using a potentiostat (VMP3, Biologic). Before each experiment, a 30‐min equilibration period was used to circulate electrolyte through the system, ensuring the membrane and electrodes were fully hydrated with their respective solutions. The anolyte composition was held constant at 0.6 M NaClO_4_, while the catholyte was varied between 0.6 M KNO_3_, KCl, NaNO_3_, or NaCl. Using K^+^ in the catholyte allowed for precise measurement of Na^+^ transport from the anolyte to the catholyte in the direction of the electric field. When Na^+^ was used in both compartments, we could compare how cation identity (either Na^+^ or K^+^) affected anion transport from the catholyte to the anolyte. After each experiment, pH measurements were taken and ion concentrations were measured using ion chromatography (Dionex, Thermo Fisher, Massachusetts). The orientation of the active layer was studied facing both the anolyte and the catholyte to determine whether the intrinsic membrane parameters fit are orientation specific or active layer specific.

### 2.3 Calculation of the Percent of Charge Carried (PCC) by Salt Ions

The PCC by all salt ions, as well as by individual anions, was calculated based on measured ion concentrations and a constant applied current density. A current density of *I*_a_ = 20 mA cm^–2^ was applied across the flow cell, requiring a constant charge flux across the membrane calculated by: $J_{c}=I_{a}/F$, where *F* is Faraday's constant. Ions moving in the direction of the electric field carry the applied current across the membrane. Using the case where the catholyte is KCl as an example, the total charge flux across the membrane can be expressed as:

(1) $$J_{c}=\left|J_{{\mathrm{Na}}^{+}}\right|+\left|J_{{\mathrm{Cl}}^{-}}\right|+\left|J_{H^{+}}\right|+\left|J_{{\mathrm{OH}}^{-}}\right|-\left|J_{K^{+}}\right|‐\left|J_{{{\mathrm{ClO}}_{4}}^{-}}\right|$$

where *J*_i_ is the flux of ion i. This equation accounts for ions such as K^+^ and ClO_4_^–^, which migrate in the direction opposite to the potential gradient; therefore, their crossover reduces the net charge carried in the desired direction and must be subtracted from the total. To express the previous equation in terms of total required charge, it can be rearranged as:

(2) $$J_{c}+\left|J_{K^{+}}\right|+\left|J_{{{\mathrm{ClO}}_{4}}^{-}}\right|=\left|J_{{\mathrm{Na}}^{+}}\right|+\left|J_{{\mathrm{Cl}}^{-}}\right|+\left|J_{H^{+}}\right|+\left|J_{{\mathrm{OH}}^{-}}\right|$$

The percentage of charge carried (PCC) by salt ions was calculated using:

(3) $${\mathrm{PCC}}_{\mathrm{salt}}=\frac{\left|J_{{\mathrm{Na}}^{+}}\right|+\left|J_{{\mathrm{Cl}}^{-}}\right|}{J_{c}+\left|J_{K^{+}}\right|+\left|J_{{{\mathrm{ClO}}_{4}}^{-}}\right|}\times 100\%$$

The percentage of charge carried by an individual ion, such as Cl^–^, was calculated as:

(4) $${\mathrm{PCC}}_{{\mathrm{Cl}}^{-}}=\frac{\left|J_{{\mathrm{Cl}}^{-}}\right|}{J_{c}+\left|J_{K^{+}}\right|+\left|J_{{{\mathrm{ClO}}_{4}}^{-}}\right|}\times 100\%$$

When KNO_3_ was used as the catholyte, $J_{{\mathrm{Cl}}^{-}}$ was replaced in Equations (1)–(4) with $J_{{{\mathrm{NO}}_{3}}^{-}}$. The fluxes of salt ions were calculated at each time point using the measured ion crossover concentrations as follows:

(5) $$J_{i}=\frac{c_{i}V}{At}$$

The electrolyte volume (*V*) at time (*t*) was multiplied by the concentration *c*_i_ of ion *i* to calculate the moles of ion in solution, which were then divided by the electrode area (*A*) and the time data point was collected. Equations (1)–(4) assume 100% Faradaic efficiency. Although some non‐Faradaic pathways may occur, previous studies show that the dominant fraction of current in saltwater electrolysis with TFC membranes arises from the intended electrochemical reactions [8]. Accordingly, this simplified calculation of PCC remains a useful tool for analyzing ion transport.

## 3 Solution‐Friction Modeling

### 3.1 Modeling Approach

Previous work validated a solution‐friction model for ion transport across TFC membranes during electrolysis, but neglected convective effects [18]. Some membrane parameters validated previously are retained here, while parameters specific to the zero‐gap electrolyzer—such as the effective diffusion through the electrodes—are fitted, as well as ion‐specific friction factors.

Ion transport was modeled using the Nernst‐Planck equation, incorporating diffusion, electromigration, and convection [18, 24]. Simulations were conducted with COMSOL Multiphysics 6.2 using the Tertiary Current Distribution electroneutrality physics to predict ion flux across the TFC membrane and carbon cloth electrodes. Ion concentrations and potentials were calculated across four unique domains (Ω). A TFC membrane is sandwiched between two carbon cloth electrodes, with the membrane support layer Ω_s_ shown facing the anode Ω_a_ and the membrane active layer Ω_m_ facing the cathode Ω_c_ (Figure 1b) [25]. Electrode thicknesses (*L*_a_ for the anode and *L*_c_ for the cathode) were assumed to match the spacer thickness (237 µm) due to the porous nature of the electrodes and compression during assembly [26, 27]. To account for the deformation of the electrodes due to compression, an effectiveness factor used to hinder diffusion through the electrodes was fit using open‐circuit diffusion data. Given that the PA active‐layer thickness (*L*_m_) is several orders of magnitude thinner than the membrane support layer, the total membrane thickness was assumed to be equal to the support layer thickness (*L*_s_ = 145 µm) [25]. The membrane and spacer thickness were measured using an outside micrometer (Mitutoyo). The active‐layer orientation relative to the electrode varied by experiment.

To minimize unknown parameters associated with the Pt/C‐coated carbon cloth electrodes, the electrodes were modeled as homogenous electrolyte domains with a single fitted diffusion factor (*ε*_e_) to mimic hindered diffusion due to the tortuous structure. Because the catalyst layer is thinly coated on the electrode surface, electrochemical reactions were assumed to occur only at the electrode interface facing the membrane [28, 29].

### 3.2 Governing Equations

The time‐dependent concentration of species *i* ,*c*_i_, with charge *z*_i_ was modeled using:

(6) $$\frac{\partial c_{i}}{\partial t}=K_{f}v_{f}\frac{dc_{i}}{dx}-\varepsilon K_{f}D_{i}\frac{d}{dx}\left(\frac{dc_{i}}{dx}+z_{i}c_{i}\frac{d\varphi }{dx}\right)+r_{i}$$

where φ is the dimensionless electric potential, *D*_i_ is the ion diffusion coefficient (taken from literature at 25°C), *ε* is the effective diffusion factor, and *K*_f_ is a frictional hinderance factor, $v_{f}$ is fluid velocity, and *r*_i_ is a reaction term. The effective diffusion coefficients were set to *ε* = 0.015 in the support layer, Ω_s_, and *ε* = 0.005 in the active layer, Ω_m_ [18, 30]. In the electrode regions, Ω_a_ and Ω_c_, the diffusion factor *ε* was fit using the open‐circuit data. The friction factor *K*_f_ represents hindered transport due to ion–ion, ion–water, or ion‐structure interactions. In the dense, membrane active layer, Ω_m_, unique friction factors *K*_f_ were fit for each ion using the open‐circuit data to isolate diffusional transport properties without the presence of a strong potential gradient and bubble generation. In the porous support layer Ω_s_ and electrode layers Ω_a_ and Ω_c_, *K*_f_ = 1.

Volume changes in the anolyte and catholyte after 6 h were measured to estimate an average water velocity across the electrolyzer stack. The change in electrolyte volume ΔV was used to calculate the volumetric flowrate (Q),using Q=ΔV/t. The time (*t*) was 6 h for all experiments. An average fluid velocity $v_{f}$ was estimated from the volumetric flow rate $v_{f}=\frac{Q}{A}$, where *A* = 4 cm^2^ is the electrode area.

Water reactions (*r*_i_) were included for H^+^ and OH^–^ using:

(7) $$r_{H^{+}}=r_{{\mathrm{OH}}^{-}}=k_{w}\left(1-\frac{c_{H^{+}}c_{{\mathrm{OH}}^{-}}}{K_{w}}\right)$$

where *k*_w_ is a rate coefficient (set large enough to maintain equilibrium), and *K*_w_ is the water equilibrium constant. All other species had *r*_i_ = 0.

### 3.3 Electroneutrality and Partitioning

The electroneutrality assumption was used to maintain charge balance across the stack according to:

(8) $$\sum_{i}z_{i}c_{i}=0$$

In the membrane active layer Ω_m_, the membrane charge density *X* with charge *z*_m_ was included:

(9) $$\sum_{i}z_{i}c_{i}+z_{m}X=0$$

Membrane charge density was set to be –400 mM when facing the catholyte solution with a pH of 13, and 10 mM when facing the anolyte solution with pH of 1 [31, 32]. These values were selected based on previous work done measuring the fixed‐charge concentrations in polyamide TFC membranes similar to those used here [32]. Previous modeling studies have shown that ion‐transport predictions in saltwater electrolysis are only weakly sensitive to reasonable variations in fixed‐charge density [18]. Because the local pH at the membrane surface adjusts much faster than the 6‐hour duration of our experiments, we assume the active‐layer charge rapidly reaches its steady‐state value. Ion partitioning into the active layer was implemented as internal boundary conditions. Donnan, steric, and dielectric partitioning controls ion transport into Ω_m_ and cause discontinuities in concentration and potential at the AL as follows:

(10) $$c_{m,i}=c_{w,i}\Phi_{i}\Phi_{D}$$

where $\Phi_{D,i}=\exp\left(-z_{i}\text{Δ}\varphi \right)$ is used to calculate Donnan partitioning due to membrane charge, and $\Phi_{i}=0.25$, based on prior fitting, captures steric, dielectric, and all other partitioning of ions [18].

### 3.4 Electrochemical Reactions and Boundary Conditions

Electrochemical species generation was modeled by imposing constant inward fluxes of H^+^ at the anode interface $J_{a,i}$ and OH^–^ at the cathode interface $J_{c,i}$ based on the applied current density:

(11) $$J_{a,H^{+}}=J_{c,{\mathrm{OH}}^{-}}=\frac{i_{a}}{F}$$

A constant current density *i*_a_ was set between the electrodes. A potential equal to 0 V was set at the cathode interface facing the electrolyte flow field as a reference point.

Boundary conditions for salt and water ions at the electrode interfaces adjacent to the flow fields were defined as ion fluxes, assuming well‐mixed 100 mL bulk electrolyte reservoirs:

(12) $$\frac{dc_{a,i}}{dt}=\frac{A}{V}\left(-J_{i}\right)+r_{i},\;\frac{dc_{c,i}}{dt}=\frac{A}{V}\left(J_{i}\right)+r_{i}$$

where *A* is the electrode area (4 cm^2^) and *V* is the electrolyte volume (100 mL). The high electrolyte recirculation rate (25 mL/min) was assumed to minimize concentration gradients along the serpentine channels. Only protons and hydroxide ions participated in the water association reaction (*r*_H+_ and *r*_OH_–), and the reaction term for all other ions was zero.

The electrolyte volume in the model was held constant at 100 mL, and measured ion transport concentrations were recalculated to represent concentrations in a 100 mL electrolyte for model fitting and validation. The experimental electrolyte volume was assumed to change linearly over time.

### 3.5 Initial Conditions

For the zero‐current case (*i*_a_ = 0), the anolyte was 0.6 M NaClO_4_, and the catholyte was 0.6 M NaNO_3_ or NaCl when comparing anion transport. When comparing cation transport, the anolyte was 0.6 M NaNO_3_, while the catholyte was 0.6 M KNO_3_. For the 20 mA cm^–2^ case, the anolyte remained 0.6 M NaClO_4_, while the catholyte was 0.6 M KNO_3_, KCl, NaNO_3_, or NaCl. The initial pH of all solutions was set to 5.7 to reflect CO_2_ absorption from air exposure. For simulations with applied current density, a 30 min zero‐current simulation was computed, and the solution from this simulation was used as the initial conditions for electrolysis experiments, mimicking the 30 min of equilibration prior to electrolysis done experimentally.

### 3.6 Model Fitting

Open‐circuit data were used to fit ion‐specific friction factors (*K*_D_) across the active layer, isolating transport behavior without the influence of gas evolution or applied potential gradients. Because multiple electrolyte compositions were studied, unique friction factors were fit for each ion rather than assigning the same value to all ions, as was done in previous solution‐friction modeling of TFC membranes during electrolysis. The open‐circuit data allowed for direct comparison of ion permeability across the membrane without the influence of an applied potential or chloride oxidation at the anode. Incorporating these differences into the model enabled isolation of convective effects on ion transport for each specific electrolyte composition and membrane configuration. The three electrolyte configurations used in the open‐circuit experiments each isolated the transport of two specific ions, allowing for iterative fitting of ion‐specific parameters across all data sets. Any observed water transport between compartments under open‐circuit conditions was incorporated into the model to ensure that the fitted parameters reflected intrinsic membrane properties rather than compensating for convective flow. Additionally, the effective diffusivity across the electrode layers (*ε*_e_) was fit using the same dataset. All model parameters are in Table S1.

## 4 Results and Discussion

### 4.1 Open‐Circuit Ion and Water Transport

**FIGURE 2:**
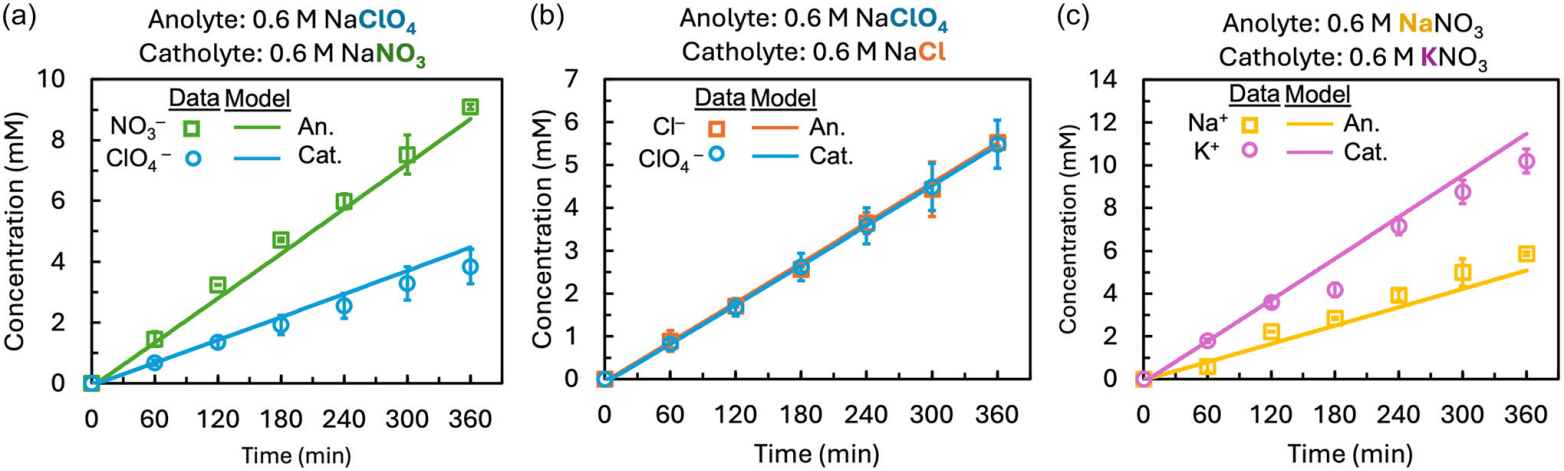
Results of ion transport across TFC membrane after 6 h of electrolyte flow through zero‐gap flow cell with no applied current density for (a) ClO_4_^–^ and NO_3_^–^ when a 0.6 M NaClO_4_ anolyte and 0.6 M NaNO_3_ catholyte were used, (b) ClO_4_^–^ and Cl^–^ when a 0.6 M NaClO_4_ anolyte and 0.6 M NaCl catholyte were used, and (c) Na^+^ and K^+^ when a 0.6 M NaNO_3_ anolyte and 0.6 M KNO_3_ catholyte were use.

Membrane‐specific friction factors were fit for each salt ion with continuity across all three experimental conditions using the open‐circuit results (Table 1 and Figure 2). The fitted friction factor for ClO_4_^–^ remained constant regardless of the catholyte used (NaNO_3_ or NaCl), suggesting its friction factor reflected intrinsic membrane properties rather than condition‐dependent effects. Similarly, the friction factors for NO_3_^–^ and Na^+^ were consistent across all conditions and matched those previously validated in a system with large electrolyte chambers [18], further supporting that these values are membrane‐specific. The electrode effectiveness factor was fit using NaClO_4_ in the anolyte and NaNO_3_ in the catholyte, and the value was set constant for the other two conditions (Table 1).

**TABLE 1:** Fitted ion‐specific friction factors and electrode diffusion effectiveness factor.

Parameter	Description	Value
$K_{D,{\mathrm{Na}}^{+}}$	Na^+^ friction factor	0.2
$K_{D,K^{+}}$	K^+^ friction factor	0.01
$K_{D,{\mathrm{Cl}}^{-}}$	Cl^–^ friction factor	0.08
$K_{D,{{\mathrm{NO}}_{3}}^{-}}$	NO_3_^–^ friction factor	0.2
$K_{D,{{\mathrm{ClO}}_{4}}^{-}}$	ClO_4_^–^ friction factor	0.011
$\varepsilon_{e}$	Electrode diffusion effectiveness factor	0.4

When the anolyte contained 0.6 M NaNO_3_, NO_3_^–^ crossed the membrane at more than twice the rate of ClO_4_^–^ (Figure 2a), reaching a final concentration of 9.1 ± 0.1 mM compared to 3.8 ± 0.6 mM for ClO_4_^–^. In the second condition, where Cl^–^ replaced NO_3_^–^ in the catholyte, both Cl^–^ and ClO_4_^–^ had nearly equal transport, with final concentrations of 5.5 ± 0.1 mM for Cl^–^ and 5.5 ± 0.4 mM for ClO_4_^–^ (Figure 2b). In the third condition, where NO_3_^–^ was used in both chambers and the cation was varied (Na^+^ in the anolyte and K^+^ in the catholyte), K^+^ transport exceeded Na^+^ by ≈70%. The final K^+^ concentration in the anolyte was 10.2 ± 1.4 mM, while Na^+^ reached 5.9 ± 0 mM in the catholyte (Figure 2c).

The observed ion transport differences between NO_3_^–^ and Cl^–^ ions are consistent with their known ion properties and previous studies [22, 23]. NO_3_^–^ permeates TFC membranes more readily than Cl^–^ due to its lower charge density and hydration energy, which reduces membrane partitioning and facilitates dehydration at the membrane interface, as demonstrated by several previous studies [33, 34, 35]. Similarly, previous work has shown that monovalent cations with larger ionic radii, such as K^+^, tend to permeate TFC membranes more easily than smaller ions like Na^+^ [36]. For example, KCl has a higher measured salt permeability than NaCl across TFC membranes [36]. This counterintuitive trend based on ion size has been attributed to the higher charge density of smaller cations and resulting stronger interactions with water molecules, which leads to larger hydrated radii and slower membrane transport [36]. Consistent with these findings, the open‐circuit results presented here show greater membrane permeation for NO_3_^–^ compared to Cl^–^ and K^+^ compared to Na^+^.

All open‐circuit experiments had measurable volume changes between compartments due to water flux through the membrane (Figure S2), despite using monovalent ions and starting with identical 0.6 M electrolyte concentrations. The driving force for osmotic gradients may have arisen from slight differences in activity coefficients or from concentration gradients formed at the charged membrane interface. Although the exact mechanism of water transport remains unclear, the observed volume changes were incorporated into the model as a water velocity term (*v*_f_). Including this transport allowed for accurate fitting of ion‐specific friction factors, as demonstrated by their consistency across all experimental conditions despite the presence of water movement.

### 4.2 Ion Transport During Electrolysis

**FIGURE 3:**
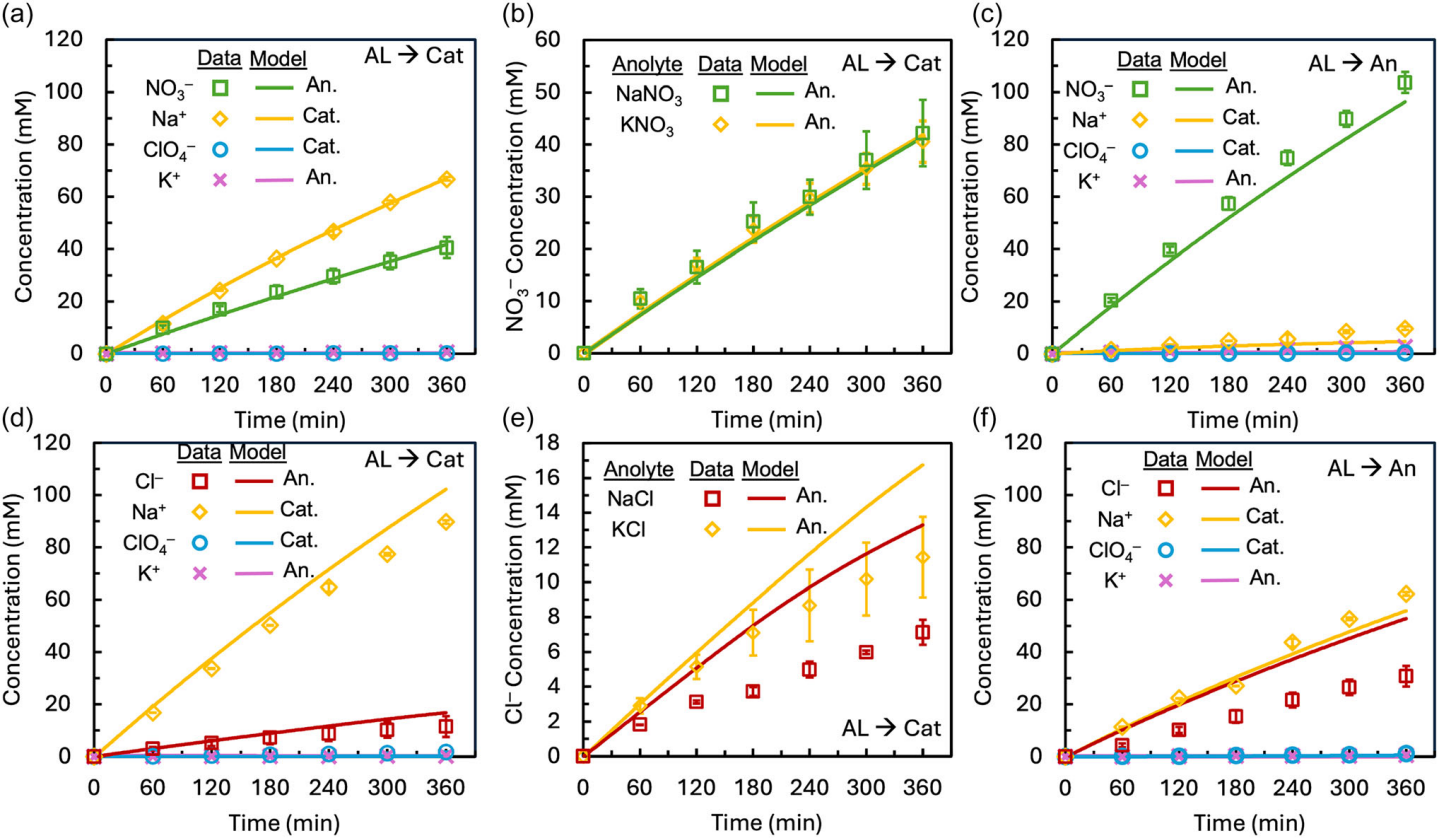
Experimental and modeled Ion transport results for (a) KNO_3_ in the catholyte with the active layer facing the cathode, (b) KNO_3_ in the catholyte with the active layer facing the anode, (c) NO_3_^–^ transport to the anolyte comparison when KNO_3_ versus NaNO_3_ was the catholyte, (d) KCl in the catholyte with the active layer facing the cathode, (e) KCl in the catholyte with the active layer facing the anode, and (f) Cl^–^ transport to the anolyte when KCl vs. NaCl was used as the catholyte.

Using the ion‐specific friction coefficients obtained from the open‐circuit experiments and incorporating the measured volume changes between electrolyte compartments, the model reasonably predicted the observed rates and extent of ion transport during water electrolysis for most ions (Figure 3). The *R* ^2^ and *p*‐values for each major ion in all conditions are reported in the supporting information (Table S2). When the active layer of the membrane was oriented toward the cathode and KNO_3_ was used as the catholyte, Na^+^ transport across the membrane exceeded that of NO_3_^–^, with the final concentrations of 67 ± 1 mM for Na^+^ and 41 ± 4 mM for NO_3_^–^ (Figure 3a). Substituting the catholyte cation from K^+^ to Na^+^—to match the open‐circuit experiments—had no effect on the measured NO_3_^–^ transport, indicating that NO_3_^–^ transport was independent of the accompanying cation (Figure 3b). When using a NaNO_3_ catholyte, the final NO_3_^–^ concentration was 42 ± 6 mM. Reorienting the membrane to have the active layer face the anode resulted in NO_3_^–^ being the dominant salt charge carrier across the membrane, while Na^+^ transport became minimal (Figure 3c). NO_3_^–^ transport increased more than 2.5×, with a final concentration of 104 ± 5 mM, and Na^+^ transport decreased 7×, with a final concentration of 10 ± 2 mM. In all three experimental configurations involving NO_3_^–^, the model successfully predicted both major salt ion transport (Figure 3a–c) and water ion transport (Figure S3a,b). These results indicate that the fitted friction factors are consistent across membrane orientations and experimental conditions, supporting their interpretation as intrinsic, membrane‐specific properties.

The model captured the overall transport trends when replacing NO_3_^–^ with Cl^–^, although there were some discrepancies between the model and experimental data. This suggests that the friction factors fit using the open‐circuit data adequately represent ion transport trends using Cl^–^ during electrolysis, despite the potential influence of Cl^–^ oxidation. With the active layer facing the cathode, Na^+^ transport across the membrane significantly exceeded that of Cl^–^, with the final concentrations of 90 ± 4 mM for Na^+^ and 11 ± 2 mM for Cl^–^ (Figure 3d). Changing the catholyte cation from K^+^ to Na^+^ resulted in a 38% decrease in Cl^–^ transport, with 7 ± 1 mM Cl^–^ observed to cross the membrane when Na^+^ was used (Figure 3e). In open‐circuit experiments, K^+^ had higher mobility across the membrane than Na^+^, so pairing Cl^–^ with a more mobile cation, K^+^, might have caused more transport of Cl^–^ across the membrane. With the membrane active layer facing the anode, Cl^–^ transport was doubled and Na^+^ transport was reduced in half, with a final Cl^–^ concentration of 31 ± 2 mM and Na^+^ concentration of 62 ± 2 (Figure 3f). When the active layer was oriented to face the anode, the model predicted nearly 70% more Cl^–^ transport across the membrane than was measured experimentally. In contrast, Na^+^ transport was accurately predicted, suggesting that either Cl^–^ oxidation occurred, or the model did not fully capture Cl^–^ transport under the changed membrane orientation.

The model accurately predicted water ion transport for all conditions, as demonstrated by the close agreement between measured and predicted pH values in both the anolyte and catholyte (Figure S3). The large applied potential gradient drives ion in its direction to cross the membrane at rates several orders of magnitude higher than ions moving against it. As a result, accurately modeling the transport of major ions was prioritized over minor ions (K^+^ and ClO_4_^–^). The concentrations of these minor ions were extremely low due to limited crossover and a 6x dilution prior to ion chromatography measurements, making precise quantification unreliable. The measured values of these minor ions are reported in the Supporting Information (Figure S4).

Orienting the AL toward the anode significantly reduced Na^+^ transport while increasing anion transport for all catholytes. This trend might be due to changes in membrane charge density due to the local pH. When facing the acidic anolyte, the polyamide AL develops a slight positive charge density (10 mM), whereas facing the cathode results in a more strongly negative membrane charge density (−400–600 mM) [31, 32]. The positive charge on the AL reduces Donnan exclusion of anions, allowing greater anion permeability when the AL faces the anode. This reduced Donnan rejection might explain the observed increase in anion transport under these conditions. Across all conditions, NO_3_^–^ transport was greater than that of Cl^–^ under otherwise identical conditions. NO_3_^–^ transport was ≈6× greater than Cl^–^ when the active layer was facing the cathode and 3× times greater when the active layer faced the anode, owing to the higher mobility of NO_3_^–^ across the active layer due to lower charge density and lower hydration energy.

**FIGURE 4:**
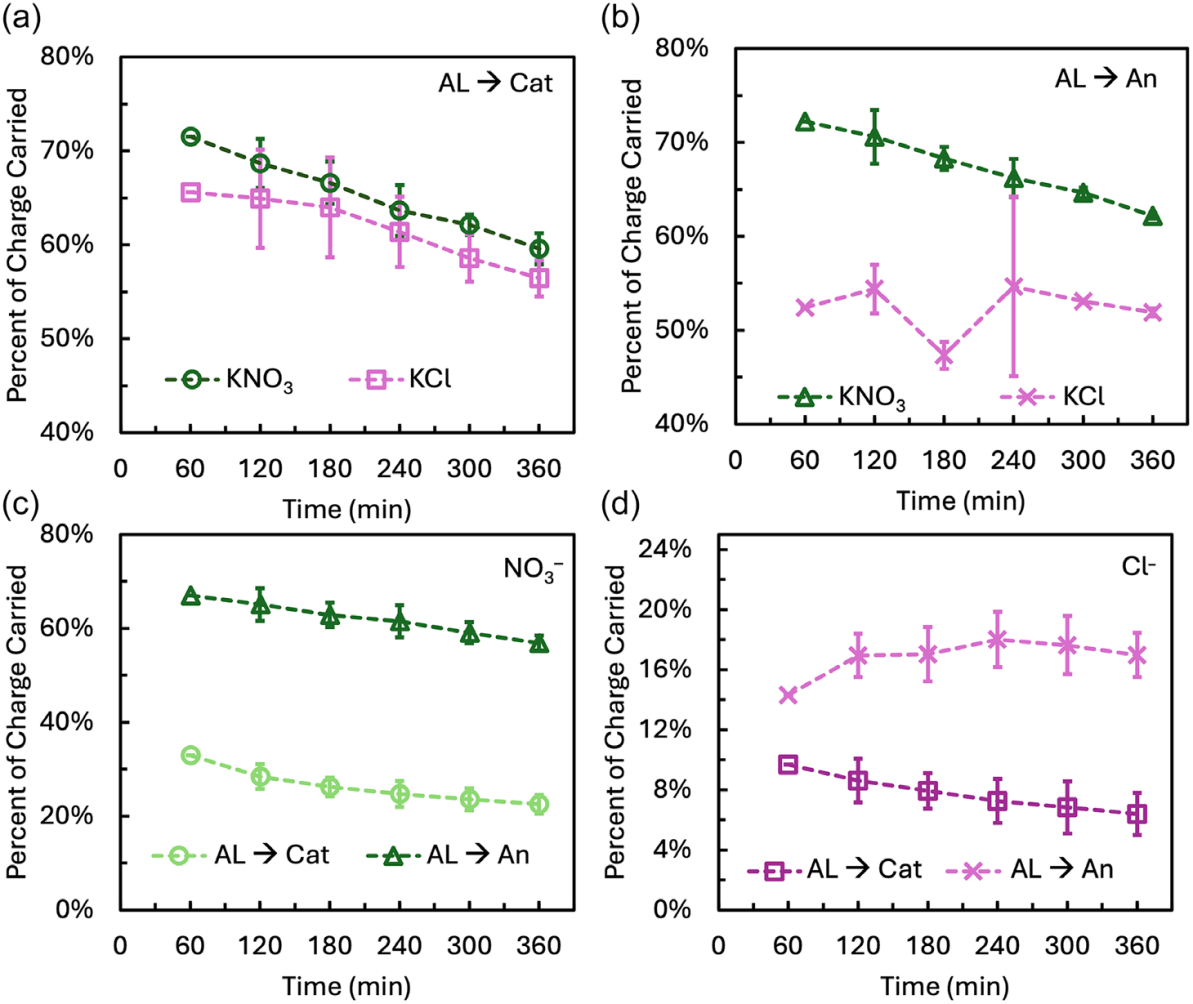
Percent of charge carried by major salt ions when using KNO_3_ or KCl as the catholyte with the active layer facing the (a) cathode and (b) anode. Percent of charge carried by (c) NO_3_^–^ with the active layer facing the cathode and anode, and (d) Cl^–^ with the active layer facing the cathode and anode. Lines are guides for the eye and do not represent a model or interpolation result.

Analyzing the PCC by salt and water ions during electrolysis provides insight into the discrepancy between model predictions and the experimental data when the membrane active layer faced the anode using a KCl catholyte. In experiments using NO_3_^–^ as the anion in the catholyte, the PCC by salt ions decreased over time as H^+^ and OH^–^ concentrations increased, allowing faster‐diffusing water ions to dominate charge transport (Figure 4a,b). This trend is consistent with previous studies and reflects the preferential use of water ions as charge carriers as their concentration builds during electrolysis [18, 19].

The PCC by salt ions was higher when using KNO_3_ compared to KCl in both AL orientations (Figure 4a,b). When the AL faced the cathode, the difference between the two electrolytes was minimal, with salt ions carrying only 2–6% less charge when using KCl (Figure 4a). When the AL faced the anode, the difference became more significant, with up to a 21% reduction in the PCC by salt ions when using KCl (Figure 4b). This greater discrepancy may be explained by Cl^–^ oxidation at the anode, which would reduce the measured Cl^–^ concentration in the anolyte and lead to an underestimation of its PCC. The larger difference observed when the AL was oriented toward the anode could reflect increased Cl^–^ oxidation due to more Cl^–^ crossover in this configuration.

A distinct trend emerged when KCl was used with the membrane facing the anode (Figure 4d). In this case, the PCC by Cl^–^ increased over time, opposite to the trend observed for other salt ions. This anomalous behavior may be explained by Cl^–^ oxidation at the anode. The observed increase in Cl^–^ PCC suggests that the rate of Cl^–^ entering the bulk electrolyte is rising over time; however, the Cl^–^ flux was expected to decrease over time due to declining electromigration and diffusion as water ion flux increases, consistent with the observed NO_3_^–^ PCC and previous studies [18, 19]. One possible explanation for this rising PCC is a decreasing Cl^–^ oxidation rate at the anode, which would reduce the extent of Cl^–^ underestimation in measurements. Cl^–^ oxidation on platinum catalyst is known to cause catalyst degradation and surface site blockage due to Cl^–^ adsorption [37], although the cause of the changing Cl^–^ oxidation selectivity in this case is unknown, and further electrochemical testing in future studies could clarify this.

This same trend of Cl^–^ increasing over time was not observed when the membrane active layer faced the cathode. Orienting the active layer toward the cathode preferentially caused the cation, Na^+^, to be the major salt ion carrier (Figure 3a,d), while orienting the active layer toward the anode caused the opposite, with NO_3_^–^ becoming the major salt charge carrier and Cl^–^ transport increasing almost threefold (Figure 3c,f). For this reason, Cl^–^ oxidation may have been more prevalent in the case when the active layer faced the anode. Cl^–^ oxidation has been well‐documented in literature as difficult to avoid during electrolysis with saltwater, even when adopting chloride oxidation prevention strategies [38]. Additionally, Pt catalyst can be more selective toward Cl^–^ oxidation over OH^–^ oxidation at low overpotentials, potentially contributing further to Cl^–^ oxidation in this case [37].

Previous studies demonstrate that salt ions carry a relatively stable percent of charge depending on the water ion availability, supporting the hypothesis that Cl^–^ oxidation is responsible for the differences between the PCC carried by salt ions when using KNO_3_ or KCl. In one experimental study, researchers modified a TFC membrane with additional size‐selective and charged layers to reduce Cl^–^ crossover by enhancing water ion transport [17]. However, every reduction in Cl^–^ transport was accompanied by a proportional increase in Na^+^ transport, suggesting that salt and water ion transport are initially decoupled due to the low concentration of water ions at early time points [17]. This trend was reinforced by a modeling study, which showed that salt ion transport across the membrane could be reduced by increasing the concentration of water ions in solution [19]. When starting from near neutral pH conditions, the water ion concentration remained too low to carry a substantial portion of charge in the initial 2 h of electrolysis, necessitating greater salt ion transport [19].

**FIGURE 5:**
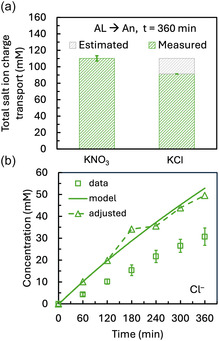
(a) Total concentration of charge carried by salt ions after 6 h of electrolysis with the AL facing the anode, using either KNO_3_ or KCl as the catholyte. Green shading indicates the measured salt ion transport in the direction of the potential gradient for each condition. The gray shaded area represents the difference between KNO_3_ and KCl cases and is used to estimate the amount of Cl^–^ oxidized during electrolysis. (b) Cl^–^ concentration in the anolyte over time: experimental measurements (square markers), model prediction (solid line), and Cl^–^ concentrations estimated in the absence of oxidation (triangle markers and dashed lines). The Cl^–^ oxidation estimate is calculated by adding the concentration difference in salt ion transport (from panel (a), gray area) calculated at each time point to the measured Cl^–^ values.

By assuming that the difference between the PCC by salt ions between KNO_3_ and KCl conditions arises from Cl^–^ oxidation, we can estimate the concentration of Cl^–^ that may have crossed the membrane without undergoing oxidation. When the membrane active layer faced the anode, the total moles of charge carried by salt ions were 110.1 ± 3.3 mM with KNO_3_ and 91.3 + 0.0 mM with KCl, yielding a difference of 18.9 mM (Figure 5a). By calculating this difference at each time point and adding it to the measured Cl^–^ concentration, we estimated that the Cl^–^ transport assuming salt ions carry a relatively constant percent of charge based on the water ion concentration (Figure 5b). This adjusted Cl^–^ profile closely aligned with model predictions, with an *R* ^2^ value of 0.98 and *p*‐value of 0.92. This improved fitting with the model suggests that salt and water ion transport are initially treated as decoupled processes by the model, with water ion transport limited by its concentration in solution.

### 4.3 Water Transport During Electrolysis

**FIGURE 6:**
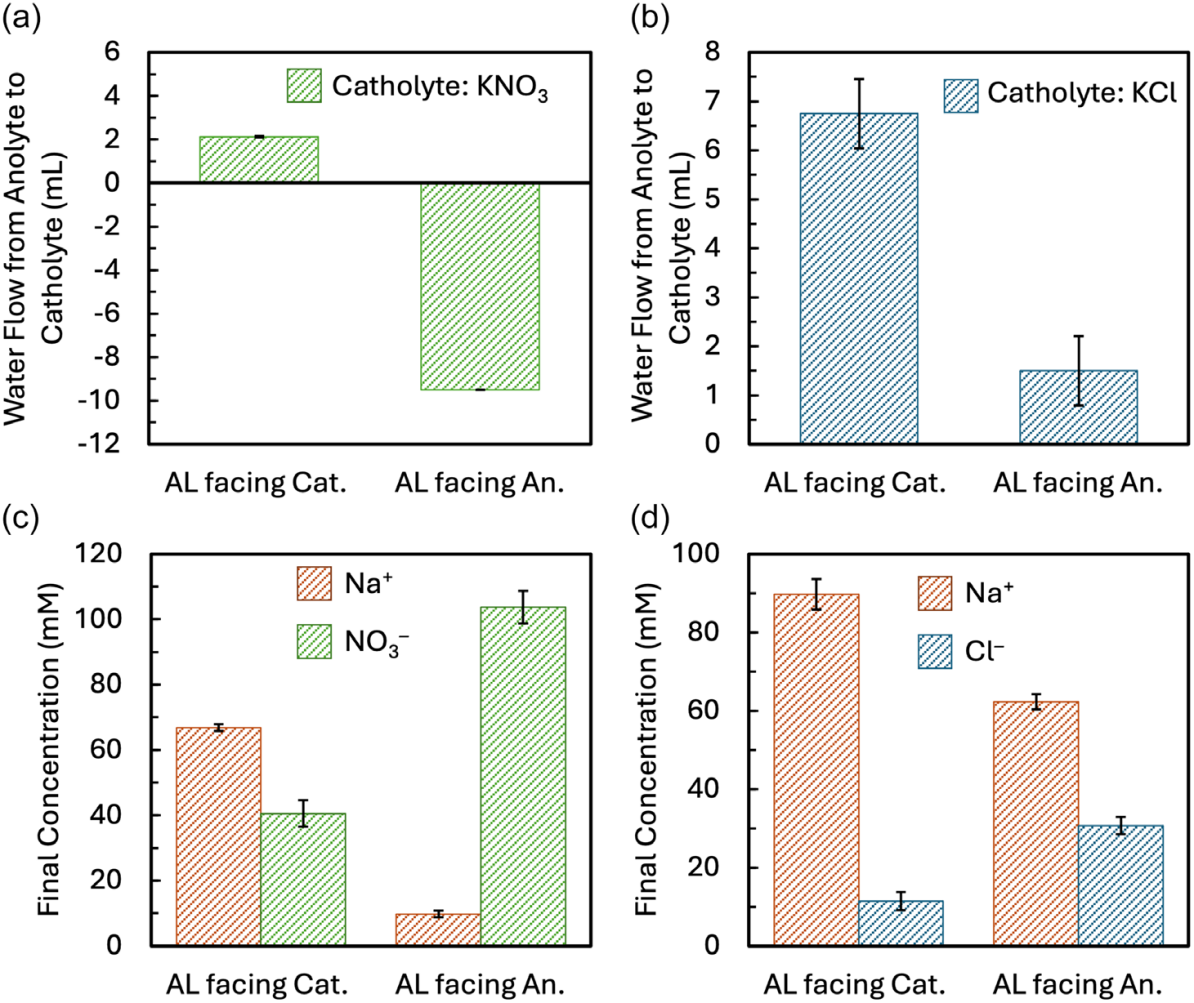
(a) Net water flow from the anolyte to the catholyte after 6 h of electrolysis with a KNO_3_ catholyte, comparing membrane AL orientation facing the cathode versus the anode. (b) Percent of total salt ion transport carried by Na^+^ and NO_3_^–^ under the same KNO_3_ catholyte conditions and AL orientations. (c) Net water flow from the anolyte to the catholyte with a KCl catholyte, comparing AL facing the cathode versus the anode. (d) Percent of total salt ion transport carried by Na^+^ or Cl^–^ with a KCl catholyte under both AL orientations. Negative values in panels (a) and (c) indicate water movement from the catholyte to the anolyte.

Significant differences in both the magnitude and direction of water transport were observed during electrolysis, depending on the catholyte composition (KNO_3_ vs. KCl) and membrane orientation (Figure 6). With KNO_3_ as the catholyte and the membrane AL facing the cathode, 2.1 ± 0.2 mL of water flowed from the anolyte to the catholyte (Figure 6a). Reversing the membrane orientation (AL facing the anode) reversed the direction of water flow, with 9.5 ± 0 mL of water moving from the catholyte to the anolyte. Switching the catholyte to KCl also influenced water transport (Figure 6b). With the AL facing the cathode, 6.8 ± 0.7 mL of water was transported from the anolyte to the catholyte. When the AL was oriented toward the anode, water transport decreased to 1.5 ± 0.7 mL, though the direction remained the same. Measured water transport during electrolysis was greater than that observed under open‐circuit conditions. Because sampling and evaporation occurred at comparable rates in both sets of experiments, these processes cannot account for the increased water loss during electrolysis. Additionally, the amount of water consumed by the electrochemical reactions—≈0.16 mL at the anode and 0.32 mL at the cathode over 6 h—is too small to explain the measured flux. Thus, the observed water transport must arise primarily from membrane‐mediated mechanisms.

To investigate the relationship between water and salt ion transport, we compared the total concentration of the major salt ions that crossed over the membrane to the direction and magnitude of fluid flow. With KNO_3_ as the catholyte and the AL facing the cathode, Na^+^ (from the anolyte) crossed over the membrane more so than NO_3_^–^, accounting for ∼60% of salt ion transport, and water migrated in the same direction as Na^+^ (Figure 6c). When the membrane orientation was reversed, NO_3_^–^ became the dominant salt ion, crossing over the membrane 10x more than Na^+^ from the catholyte to the anolyte (Figure 6c). This switch in major ion transport corresponded with the observed reversal in water flow, suggesting a relationship between the dominant salt charge carrier and water movement. Similar trends were observed when using KCl as the catholyte (Figure 6d). Regardless of membrane orientation, Na^+^ consistently transported across the membrane more than Cl^–^, and water flowed in the same direction as Na^+^ in both conditions. However, when the AL faced the anode, the total Na^+^ crossover decreased while Cl^–^ crossover increased, and a corresponding reduction in the magnitude of water transport toward the anode was observed (Figure 6d). These results support the observation that water transport is correlated to the direction and magnitude of the dominant salt ion flux, suggesting electroosmotic drag may be one of the mechanisms responsible for the observed water transport. Other mechanisms contributing to the water flux across the membrane could include bubble generation, imbalances in activity coefficients between the anolyte and catholyte, or slight pressure differences across the membranes.

**FIGURE 7:**
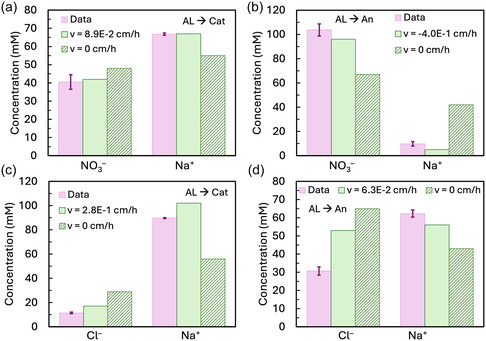
Comparison of measured and modeled total major salt ion transport (Na^+^, NO_3_^–^, or Cl^–^) with or without convection. (a) 0.6 M NaNO_3_ catholyte, membrane AL facing the cathode; (b) 0.6 M NaNO_3_ catholyte, AL facing the anode; (c) 0.6 M NaCl catholyte, AL facing the anode; (d) 0.6 M NaCl catholyte, AL facing the cathode. Model predictions are shown for both the case where *v*_f_ is set to the experimentally determined value and where *v*_f_ is set to zero.

To evaluate the role of convective water flow on ion transport, we set the fluid velocity term *v*_f_ to zero in the model. By modeling ion transport with and without convection, we could assess how neglecting convection impacts model agreement with data under the different electrolyte and membrane orientation conditions. When using KNO_3_ as the catholyte with the AL facing the cathode, neglecting convection resulted in a 14% overprediction of NO_3_^–^ transport and a 18% underprediction of Na^+^ transport (Figure 7a). Reversing the membrane orientation to face the anode in the absence of water flow led to even greater discrepancies, with the model predicting 30% less NO_3_^–^ transport and 740% more Na^+^ transport (Figure 7b). Without convection, the model predicted 42 mM of transport, while it predicted 5 mM of transport with convection, closer to the true value of 10 mM (Figure 7b). For experiments using KCl as the catholyte, the effect of convection was more pronounced when the AL faced the cathode (Figure 7c). In this case, neglecting convection led to a 71% increase in predicted Cl^–^ transport and a 45% decrease in predicted Na^+^ transport. When the AL was oriented toward the anode, the model predicted 23% more Cl^–^ transport and 23% less Na^+^, resulting in a final concentration of 65 mM for Cl^–^ and 43 mM for Na^+^ (Figure 7d).

When salt ions were transported at a more similar rate, rather than one salt ion dominating charge transport, water movement across the membrane was reduced, and convection had a smaller impact on the model's salt ion transport predictions. In contrast, when a single salt ion carried most of the charge, large volume changes were measured in the electrolytes due to water flux, making convection's influence on ion transport more pronounced. These results highlight the interconnected nature of ion and water transport in a zero‐gap flow cell during saltwater electrolysis with a TFC membrane.

## 5 Conclusions

By fitting unique ion friction coefficients and incorporating measured water transport into a solution‐friction transport model, we isolated the contribution of convection on ion transport during saltwater electrolysis in a zero‐gap flow cell. The different electrolyte compositions studied exhibited distinct transport behaviors across the membrane, with NO_3_^–^ permeating more readily than Cl^–^ in both open‐circuit and electrolysis conditions. Membrane orientation also played a critical role in determining which salt ion—cation (Na^+^) or anion (NO_3_^–^ or Cl^–^)—dominated transport across the membrane. The direction and magnitude of salt ion transport were strongly correlated with the direction and extent of water transport, highlighting the interconnected nature of water and ion transport during zero‐gap electrolysis. The results have important implications for reducing Cl^–^ crossover to the anode, where it can oxidize to form chlorine or other reactive chlorine species that damage electrolyzer components. Orienting the membrane with its active layer facing the cathode was found to be the most effective in reducing chloride transport, most likely due to the larger Donnan partitioning from the negatively charged active layer. In this configuration, water moved from the anolyte to the catholyte, which may further suppress Cl^–^ transport. However, the loss of water from the anolyte is not desirable as this would require makeup water to replace both that lost to water oxidation as well as flow into the catholyte. Limiting water transport from the catholyte to the anolyte by osmotically balancing the electrolyte solutions can help reduce Cl^–^ crossover driven by electroosmotic drag. Moreover, decreasing the osmotic pressure in the anolyte might enable water flow from the catholyte into the anolyte, but this might enhance Cl^–^ transport. Additional research should focus on addressing water flow and ion transport through osmotic balancing of the separate electrolytes.

In typical RO and NF systems, water transport across the membrane is small enough that convection is not one of the main mechanisms driving ion transport across the membrane. Instead, diffusion drives ion transport according to the solution‐diffusion mechanisms [24]. In typical water electrolysis systems, water transport occurs more readily across the membrane due to electroosmotic drag, bubble generation, and other mechanisms, causing convection to play a larger role in ion transport [20]. For saltwater electrolysis with TFC membranes, further experiments are required to fully understand the mechanistic reason behind water flux across the membrane. Adapting techniques for measuring electroosmotic drag coefficients to TFC membranes could also help in the future to mechanistically validate the source of water flux across the membrane.

## Supporting Information

Additional supporting information can be found online in the Supporting Information section. The authors have cited additional references within the Supporting Information [18, 19, 25, 30, 32, 39, 40, 41, 42, 43]. **Supporting** **Fig. S1** **:** (a) Picture of zero‐gap flow cell set up with peristaltic pump and electrolyte reservoirs. (b) picture of the serpentine flow channels on the graphite (left) and titanium (right) current collectors. **Supporting** **Fig. S2** **:** Flow from anolyte to catholyte during open‐circuit experiments. **Supporting** **Fig. S3** **:** Minor ion transport across the membrane during electrolysis with (a) KNO_3_ catholyte and the AL facing the cathode, (b) KNO_3_ catholyte and AL facing the anode, (c) KCl catholyte and the AL facing the cathode, and (d) KCl catholyte and the AL facing the anode. **Supporting** **Fig. S4** **:** Measured and predicted pH values in the anolyte and catholyte for (a) a KNO_3_ catholyte with the AL facing the cathode, (b) a KNO_3_ catholyte with the active layer facing the anode, (c) a KCl catholyte with the active layer facing the cathode, (d) a KCl catholyte with the active layer facing the anode. The anolyte was NaClO_4_ for all the four cases. **Supporting** **Table S1:** Parameters used in solution friction model. **Supporting** **Table S2:** *R* ^2^ and *p*‐values for experimental data and model predictions for major ions.

## Funding

This research was funded by National Science Foundation grant CBET‐2027552 and Penn State University through the Stan and Flora Kappe endowment (96840).

## Conflicts of Interest

The authors declare no conflicts of interest.

## Supporting information

Supplementary Material
