# Supplementary material for: Modeling Zero‐Gap Saltwater Electrolysis With Advective Flow Through a Thin‐Film Composite Membrane
Source: ChemSusChem. 2026 Feb 8;19(3):e202501310. doi: 10.1002/cssc.202501310 (PMC12883095; doi:10.1002/cssc.202501310)
Supplement: Supplementary file 1 — Supplementary Material [file CSSC-19-e202501310-s001.pdf]

Supplementary Information

Modeling zero-gap saltwater electrolysis with advective flow through a thin-film composite membrane

Rachel F. Taylor,[b] Chenghan Xie[a], Bin Bian[a], Amir Akbari[b], and Bruce E. Logan*[a]

[a] Professor Bruce E Logan, Chenghan Xie, Bin Bian
Department of Civil and Environmental Engineering
Pennsylvania State University
University Park, PA, USA
bel3@psu.edu

[b] Rachel Taylor, Amir Akbari
Department of Chemical Engineering
Pennsylvania State University
University Park, PA, USA

S1. Zero-gap flow cell apparatus

Ion and water transport were studied using a zero-gap flow cell configuration (Figure S1). In this setup, two red end plates secured the membrane-electrode assembly. The membrane was positioned between two carbon cloth electrodes coated with Pt/C catalyst. On the anode side, a titanium current collector with a serpentine flow channel was placed adjacent to the electrode, while on the cathode side, a graphite current collector with the same channel design was used. Electrolytes were circulated through the cell using a peristaltic pump at a flow rate of 25 mL/min, with 100 mL reservoirs serving as the electrolyte source for each side.

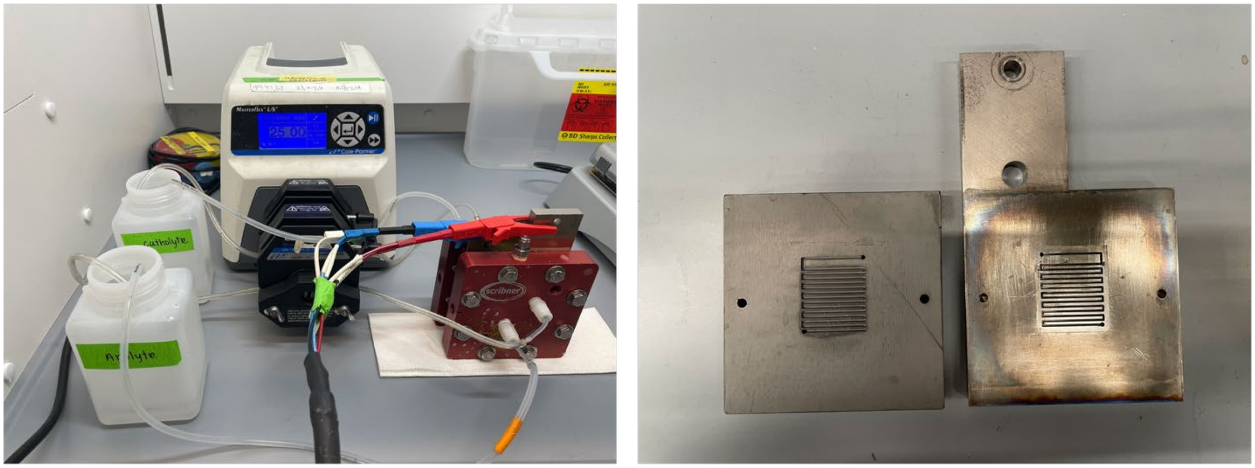

Figure S1. (a) Picture of zero-gap flow cell set up with peristaltic pump and electrolyte reservoirs. (b) picture of the serpentine flow channels on the graphite (left) and titanium (right) current collectors.

S2. Parameters used for solution-friction model

Table S1. Parameters used in solution friction model

Parameter	Description	Value	Unit	Source/Notes
		Geometry		
A	Electrode Area	4	cm^2	measured
V	Electrolyte Volume	100	mL	measured
L_a, L_c	Electrode Thickness	237	μm	measured
L_s	Support layer thickness	145	μm	measured
L_m	Active layer thickness	150	nm	[1]
		Mass transport		
D_i	Diffusion coefficients in water		$m^2 s^{-1}$	20 °C
	Na^+	1.3×10^{-9}		[2]
	NO_3^-	1.7×10^{-9}		[3]
	K^+	2.0×10^{-9}		[2]
	ClO_4^-	1.8×10^{-9}		[4]
	H^+	9.3×10^{-9}		[5]

	OH^-	5.3×10^{-9}		[2]
$K_{D,i}$	Friction coefficients		-	
	Na^+	0.2		fitted
	NO_3^-	0.2		fitted
	K^+	0.01		fitted
	ClO_4^-	0.011		fitted
	H^+	0.2		[6]
	OH^-	0.2		[6]
ε_e	Electrode diffusion effectiveness factor	0.4	-	fitted
ε_a	Membrane active layer diffusion effectiveness factor	0.005	-	[6, 7]
ε_s	Membrane support layer diffusion effectiveness factor	0.015	-	[6]
Φ_i	Steric and dielectric partitioning		-	
	Na^+, NO_3^-, K^+,ClO_4^-	0.25		[6]
	H^+, OH^-	1		[8]
X_a	Membrane charge density facing anolyte	10	mM	[9]
X_c	Membrane charge density facing catholyte	400	mM	[9]
$z_{m,a}$	Membrane charge facing anolyte	1	-	[9]
$z_{m,c}$	Membrane charge facing catholyte	-1	-	[9]
Aqueous equilibria				
K_w	Water equilibrium constant	1×10^{-14}	mol^2 L^{-2}	
k_w	Water association rate constant	1×10^{-4}	mol m^{-3} s^{-1}	arbitrary value ensuring near equilibrium
Operation				
i_a	Applied current density	20	mA cm^{-2}	measured
$c_{0,a}$, $c_{0,c}$	Initial concentrations anolyte/ catholyte	600	mM	measured
pH_0	Initial pH in anolyte / catholyte	5.7	-	measured
Others				
T	Temperature	293.15	K	measured
R	Gas constant	8.3145	J mol^{-1} K^{-1}	
F	Faraday constant	96485	C mol^{-1}	

S3. Water transport during open-circuit experiments

During open-circuit experiments, measurable water transport was observed over a 6-hour period (Figure S2). To account for this, the observed water transport was incorporated into the model as a fluid velocity term (v_f), ensuring that the friction factors used in the solution friction model remained independent of the measured water transport.

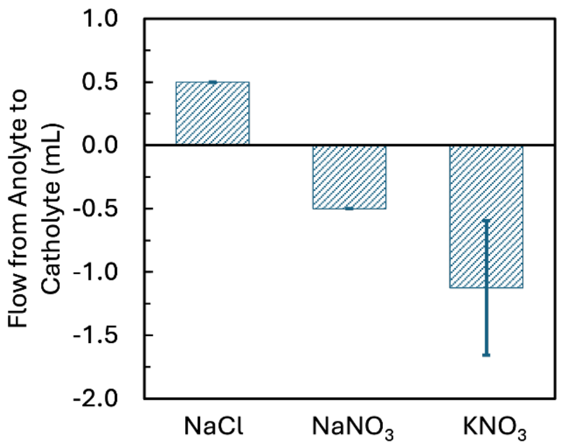

Figure 2. Flow from anolyte to catholyte during open-circuit experiments.

S4. R^2 and p-values for experimental data and model predictions

Table S2. R^2 and p-values for experimental data and model predictions for major ions

Catholyte, AL Orientation	Ion	R^2	p-value
KNO_3, Cat	Na^+	1	0.17
	NO_3^-	0.99	0.12
KNO_3, An	Na^+	0.25	0.02
	NO_3^-	0.97	0.004
KCl, Cat	Na^+	0.95	0.01
	Cl^-	0.43	0.03
KCl, An	Na^+	0.97	0.15
	NO_3^-	-0.77	0.006

S4. Minor ion and water ion transport during electrolysis and model fitting

Due to the large applied potential, salt ions moving in the direction of the electric field were transported across the reactor at rates several orders of magnitude higher than those of the minor ions (that moved against the direction of the electric field). As a result, fitting the model to the major salt ions (Na^+, NO_3-, Cl-) was prioritized over fitting to the minor ions. While the model did not accurately capture the behavior of minor ions, their crossover concentration at the end of the electrolysis experiments was typically less than 3 mM, making this discrepancy acceptable (Figure S3). Additionally, the samples were diluted 6x before they were measured using ion chromatography, so the concentrations of minor ions being measured were 6x lower than what is reported here, and the accuracy of the ion chromatography measurements cannot be guaranteed at those low concentrations.

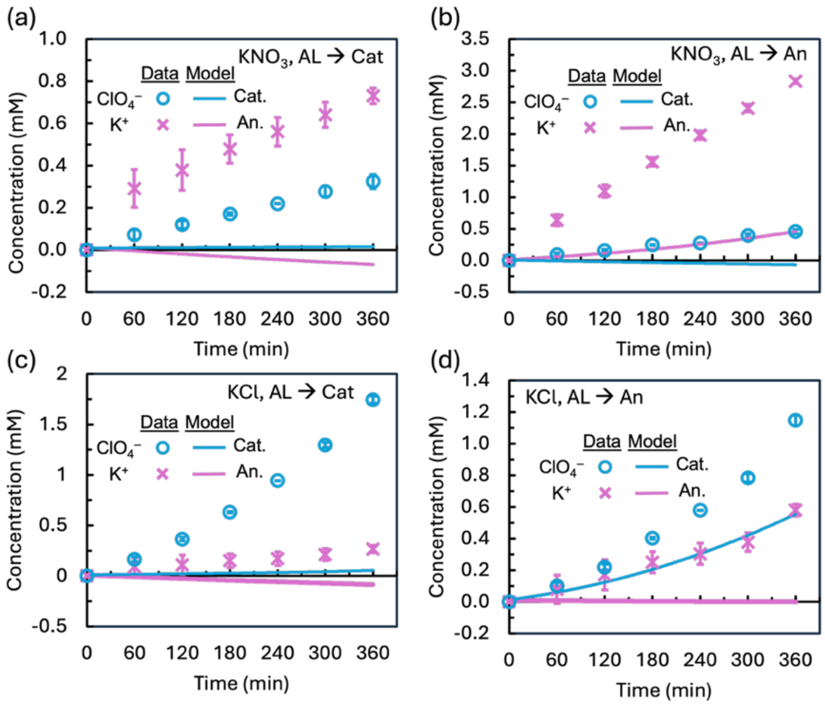

Figure S3. Minor ion transport across the membrane during electrolysis with (a) KNO_3 catholyte and the AL facing the cathode, (b) KNO_3 catholyte and AL facing the anode, (c) KCl catholyte and the AL facing the cathode, and (d) KCl catholyte and the AL facing the anode.

Water ion transport was also fit using measured pH data from the electrolysis experiments. (Figure S4). The model accurately predicted water ion transport in all cases with the fitting parameters and measured water velocities. The friction and partition coefficients for water ions from the previous modeling studies were retained here [6, 10].

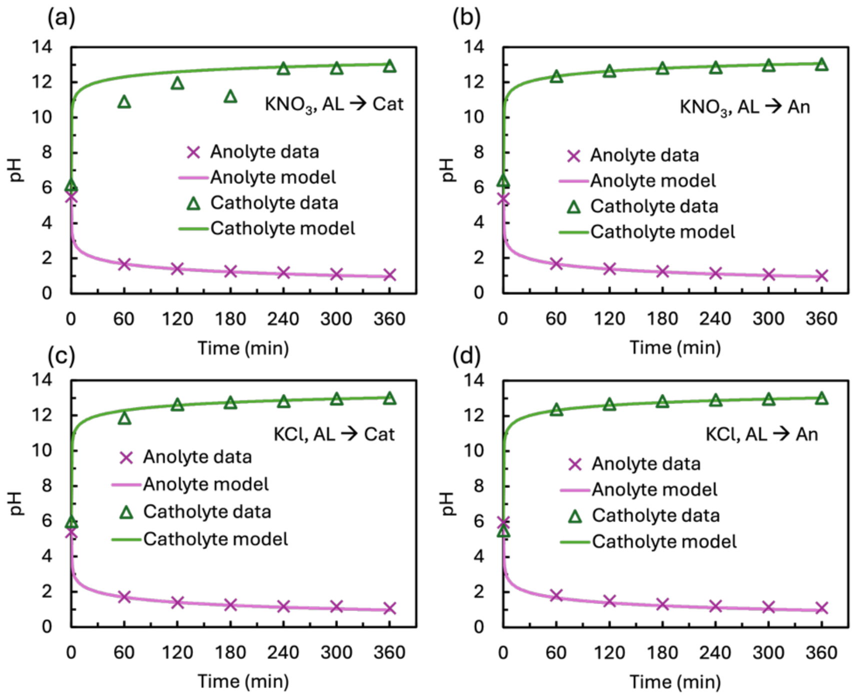

Figure S4. Measured and predicted pH values in the anolyte and catholyte for (a) a KNO_3 catholyte with the AL facing the cathode, (b) a KNO_3 catholyte with the active layer facing the anode, (c) a KCl catholyte with the active layer facing the cathode, (d) a KCl catholyte with the active layer facing the anode. The anolyte was $NaClO_4$ for all the four cases.

Works Cited

[1] K. H. Chu, J. S. Mang, J. Lim, S. Hong, M.-H. Hwang, "Variation of free volume and thickness by high pressure applied on thin film composite reverse osmosis membrane" *Desalination* **2021**, *520*, 115365.

[2] E. Samson, J. Marchand, K. A. Snyder, "Calculation of ionic diffusion coefficients on the basis of migration test results" *Mater. Struct.* **2003**, *36*, 156-165.

[3] J. U. Kreft, C. Picioreanu, J. W. Wimpenny, M. C. van Loosdrecht, "Individual-based modelling of biofilms" *Microbiology* **2001**, *147*, 2897-2912.

[4] S. Heil, M. Holz, T. Kastner, H. Weingärtner, "Self-diffusion of the Perchlorate Ion in Aqueous Electrolyte Solutions Measured by 35-Cl NMR Spin-Echo experiments" *J. Chem. Soc. Faraday Trans.* **1995**, *91*, 1877-1880.

[5] N. Agmon, "The Grotthuss mechanism" *Chem. Phys. Lett.* **1995**, *244*, 456-462.

[6] R. F. Taylor, X. Zhou, C. Xie, F. Martinez, X. Zhang, B. Blankert, C. Picioreanu, B. E. Logan, "Modeling ion transport across thin-film composite membranes during saltwater electrolysis" *Environ. Sci. Technol.* **2024**, *58*, 10969-10978.

[7] T. E. Culp, Y. X. Shen, M. Geitner, M. Paul, A. Roy, M. J. Behr, S. Rosenberg, J. Gu, M. Kumar, E. D. Gomez, "Electron tomography reveals details of the internal microstructure of desalination membranes" *Proc. Natl. Acad. Sci. USA* **2018**, *115*, 8694-8699.

[8] L. Zhang, H. V. M. Hamelers, P. M. Biesheuvel, "Modeling permeate pH in RO membranes by the extended Donnan steric model" *J. Membr. Sci.* **2020**, *613*.

[9] O. Coronell, M. I. González, B. J. Mariñas, D. G. Cahill, "Ionization behavior, stoichiometry of association, and accessibility of functional groups in the active layers of reverse osmosis and nanofiltration membranes" *Environ. Sci. Technol.* **2010**, *44*, 6808-6814.

[10] R. F. Taylor, F. Martinez-Jimenez, B. E. Logan, "Tuning initial pH to decrease salt ion transport in saltwater electrolysis" *Electrochem. Commun.* **2025**, *171*, 107858.
